# CD147 confers temozolomide resistance of glioma cells via the regulation of β-TrCP/Nrf2 pathway

**DOI:** 10.7150/ijbs.60894

**Published:** 2021-07-13

**Authors:** Xin Bu, Xuan Qu, Kai Guo, Xiangliang Meng, Xing Yang, Qike Huang, Wenjie Dou, Lin Feng, Xinxin Wei, Jiwei Gao, Wei Sun, Min Chao, Liying Han, Yaqin Hu, Liangliang Shen, Jian Zhang, Liang Wang

**Affiliations:** 1 The State Key Laboratory of Cancer Biology, Department of Biochemistry and Molecular Biology, The Fourth Military Medical University, Xi'an, 710032, China.; 2 Shaanxi University of Chinese Medicine, Xianyang, 712046, China.; 3 Department of Burns and Cutaneous Surgery, Xijing Hospital, The Fourth Military Medical University, Xi'an, 710032, China.; 4 JingKai NO. 3 Middle School, Xi'an, 710200, China.; 5 The 3rd Department of Hepatic Surgery, Eastern Hepatobiliary Hospital, Naval Medical University, Shanghai, 200438, China.; 6 Department of Plastic and Reconstructive Surgery, Xijing Hospital, Fourth Military Medical University, Xi'an, 710032, China.; 7 Basic Medical College, Jiamusi University, Jiamusi, 154002, China.; 8 Department of Oral and Maxillofacial Surgery, Hospital of Stomatology, Jiamusi University, Jiamusi, 154002, China.; 9 Department of General Surgery, Tangdu Hospital, The Fourth Military Medical University, Xi'an, 710038, China.; 10 Department of Anorectal, the General Hospital of PLA Tibet Military Area Command, Lhasa, 850007, China.; 11 Department of Neurosurgery, Tangdu Hospital, The Fourth Military Medical University, Xi'an, 710038, China.

**Keywords:** CD147, Temozolomide, Glioma, β-TrCP, Nrf2

## Abstract

**Background:** Drug resistance is one of the biggest challenges in cancer therapy. temozolomide (TMZ) represents the most important chemotherapeutic option for glioma treatment. However, the therapeutic efficacy of TMZ remains very limited due to its frequent resistance in glioma, and the underlying mechanisms were not fully addressed. Herein, we demonstrate that the elevated expression of CD147 contributes to TMZ resistance in glioma cells, potentially through the post-translational regulation of Nrf2 expression.

**Methods:** Cell-based assays of CD147 triggered drug resistance were performed through Edu-incorporation assay, CCK8 assay, TUNEL staining assay and flow cytometric assay. Luciferase reporter assay, protein stability related assays, co-immunoprecipitation assay were used to determine CD147 induction of Nrf2 expression through β-TrCP dependent ubiquitin system. Finally, the effect of the CD147/Nrf2 signaling on glioma progression and TMZ resistance were evaluated by functional experiments and clinical samples.

**Results:** Based on the analysis of clinical glioma tissues, CD147 is highly expressed in glioma tissues and positively associated with tumor malignancy. Suppression of CD147 expression increased the inhibitory effect of TMZ on cell survival in both U251 and T98G cells, whereas the gain of CD147 function blocked TMZ-induced ROS production and cell death. Mechanistic study indicates that CD147 inhibited GSK3β/β-TrCP-dependent Nrf2 degradation by promoting Akt activation, and subsequently increased Nrf2-mediated anti-oxidant gene expressions. Supporting the biological significance, the reciprocal relationship between CD147 and Nrf2 was observed in glioma tissues, and associated with patient outcome.

**Conclusions:** Our data provide the first evidence that glioma resistance to TMZ is potentially due to the activation of CD147/Nrf2 axis. CD147 promotes Nrf2 stability through the suppression of GSK3β/β-TrCP dependent Nrf2 protein degradation, which results in the ablation of TMZ induced ROS production. As such, we point out that targeting CD147/Nrf2 axis may provide a new strategy for the treatment of TMZ resistant gliomas.

## Introduction

Glioma, characterized by its undesirable prognosis and poor survival rate, is a serious threat to human health 1, 2. Due to the lack of tumor markers, early diagnosis is not effective, resulting in the poor outcome. As a first-line chemotherapy drug for glioma, temozolomide (TMZ) has greatly improved the efficacy of glioma chemotherapy, but drug resistance often occurs 3, 4. Thus, it is of great significance to seek new therapeutic targets or signal pathways for in-depth understanding of the drug resistance mechanism and clinical treatment of glioma.

Nuclear factor E2-related factor 2 (Nrf2) is an important transcription factor mediating the cellular defense response. Nrf2 identifies and transcriptionally activates genes with anti-oxidative response element (ARE) response elements on the promoter, and is involved in many biological processes, such as redox homeostasis, tumor development, cancer stem cell activity and drug resistance 5-7. Nrf2 protein levels were affected by a variety of post-translational modifications. Kelch-like ECH-associated protein 1 (KEAP1) is a classical factor regulating Nrf2, and forms a dimer complex with Nrf2 through its C-terminal BTB/POZ region, which results in the degradation mediated by E3 ligase Cullin-3 8. Nrf2 is also regulated by GSK3β and SCF/β-TrCP (β-transducin repeats-containing proteins). GSK3β promotes SCF/β-TrCP-specific ubiquitination and degradation by phosphorylation of serine residues in the Neh6 domain of Nrf2, and also inhibit its nuclear translocation 9. The high levels of Nrf2 has been observed in a variety of cancers including glioma, and predominantly in glioma stem cells 7, 10.

CD147 is a transmembrane glycoprotein composed of 269 amino acids, including the extracellular N-terminal immunoglobulin region, the transmembrane region, and the C-terminal intracellular region. CD147 can interact with multiple proteins, such as monocarborxylat transporter, intergrins and matrix metallopeptidase, which are associated with the tumor proliferation and progression 11-13. However, whether and how CD147 contributes to glioma cell survival and drug resistance were largely unknown. In this study, we identified that CD147 is highly expressed in glioma tissues, and associated with tumor grade and prognosis. The high levels of CD147 expression increases the resistance of glioma cells to TMZ treatment, potentially through the elimination of intracellular reactive oxygen species (ROS) production. Mechanistic study showed that CD147 induces Nrf2 expression through the blockage of β-TrCP mediated Nrf2 protein degradation by regulating Akt/GSK3β pathway. Thus, targeting CD147 may provide new target for the treatment of TMZ resistant glioma.

## Results

### CD147 is highly expressed in glioma tissues and associated with tumor malignancy

**Figure 1:**
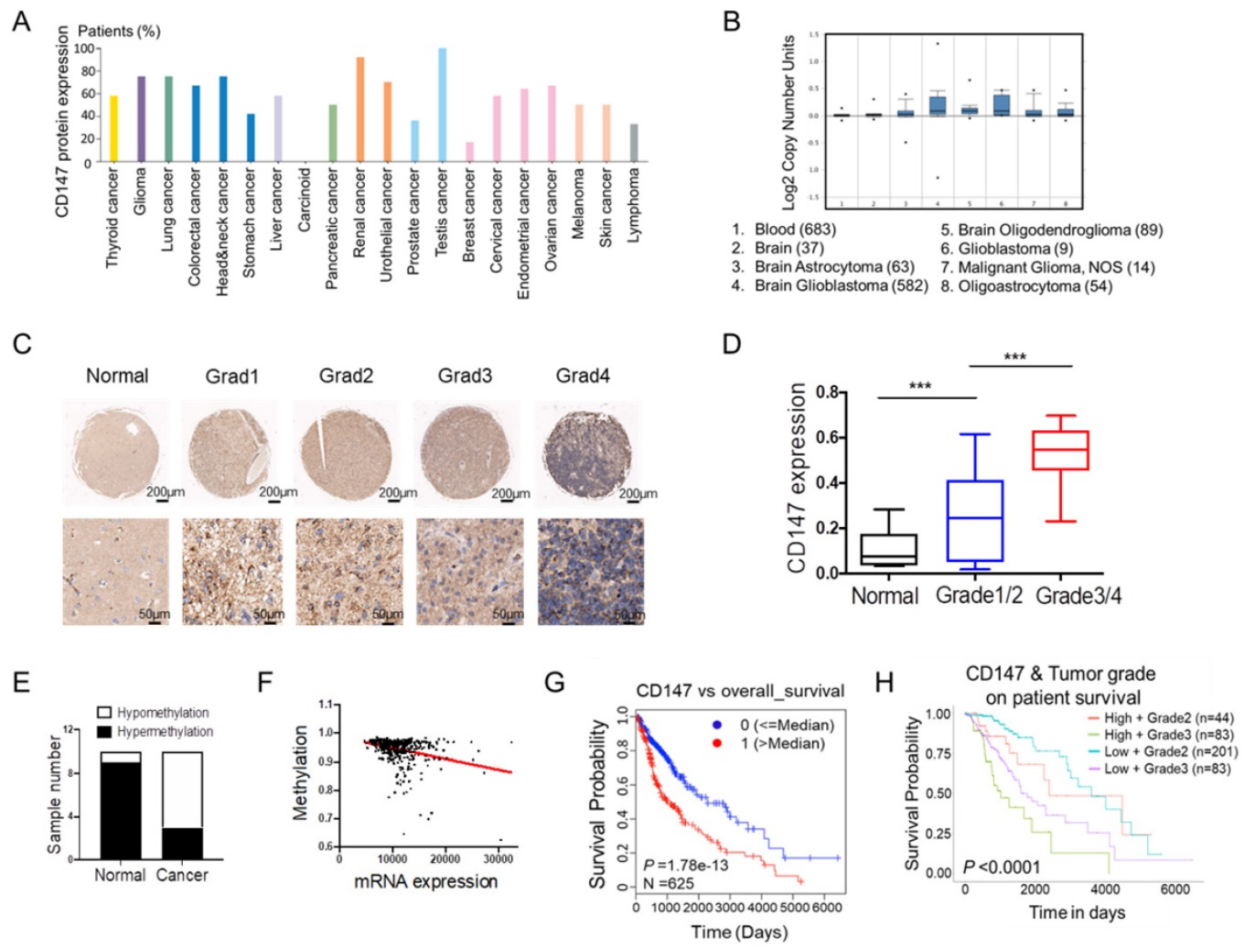
** CD147 is highly expressed in glioma tissues and associated with tumor malignancy** (A-B) Silico analysis of glioma tissues of the multidimensional data set from The Cancer Genome Atlas (TCGA) PROTEINATLAS data set. CD147 protein expression and copy numbers were determined in various types of tumor sections respectively. (C-D) Immunohistochemistry staining of CD147 in human normal and glioma tissues with different grade (C) and the statistical analysis (D). (E) The methylation status of the CD147 promoter was analyzed by methylation-specific PCR in each of the 20 normal and glioma tissues. The white and black colors represent hypomethylation and hypermethylation, respectively. (F) The correlation between the TCGA expression and methylation data for CD147 in glioma tissues. (G-H) Clinical outcomes for the gene expression patterns given at the top of each panel by UALCAN analysis.

To fully address the clinical significance of CD147 in patients with tumors, we firstly analyzed the expression levels of CD147 in various types of cancers. Based on the data from THRA (the human protein atlas) datasets, we identified CD147 protein expressions and copy numbers are significantly increased in numerous cancer tissues including glioma (Figure 1A and 1B). To confirm this data, we determined CD147 expression in in glioma and normal brain tissues from 70 patients. Immunohistochemical and statistical analysis showed that CD147 was broadly highly expressed in glioma tissues and positively associated with tumor malignancy (Figure 1C and 1D). The high levels of CD147 in glioma might be due to the hypomethylation of CD147 promoter, which were identified by both methylation-specific PCR in glioma samples and the multidimensional data set from The Cancer Genome Atlas (TCGA) (Figure 1E and 1F). Intriguingly, high CD147 expression correlated with decreased survival (Figure 1G), which was more pronounced in patients with high grade tumors (Figure 1H). Thus, CD147 might be a target for the treatment of malignant glioma.

### CD147 contributes to the resistance to TMZ treatment via the elimination of intracellular ROS

**Figure 2:**
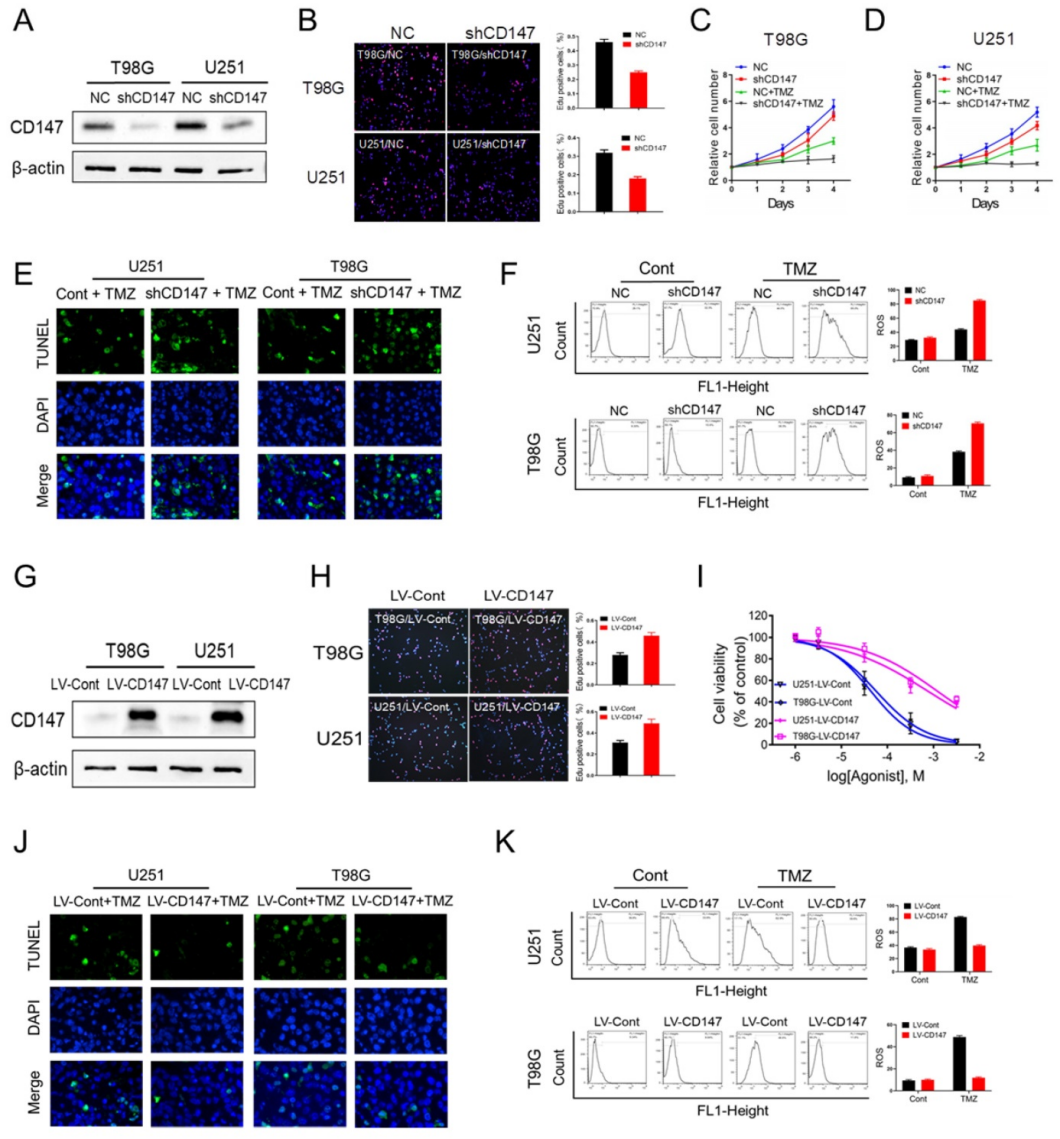
** CD147 contributes to the resistance to TMZ treatment via the elimination of intracellular ROS** (A-F) We reduced CD147 levels in U251 and T98G cells using lentivirus expressing CD147 shRNA, and then treated with 50 μM TMZ as indicated. (A) Identification of CD147 protein levels. (B-D) Cell viabilities were determined by Edu incorporation (B) and CCK8 assay (C-D) in indicated cells. (E) TUNEL staining was performed to determine cell apoptosis. (F) Relative ROS production was determined by Flow cytometry. (G-K) CD147 was overexpressed in U251 and T98G cells with CD147 overexpressing lentivirus, and then treated with TMZ as indicated. (G) Identification of CD147 protein levels. (H-K) cell viabilities (H-I), apoptosis (J) and ROS production (K) were determined, respectively.

To investigate whether CD147 contributes to glioma cell survival and malignant phenotype generation, we generated CD147 stable knockdown or overexpressing glioma cell line U251 and T98G. Our data demonstrated that suppression of CD147 expression by shRNA dramatically decreased glioma cell growth, which were confirmed by either CCK8 or Edu incorporation assay (Figure 2A-2D). Furthermore, CD147 reduction increased the inhibitory effect of TMZ on cell survival in both U251 and T98G cells (Figure 2C and 2D). Thus, CD147 may play a key role in regulation of TMZ resistance of glioma cells. Supporting this idea, TMZ treatment lead to the death of glioma cells, and the increased cell death were pronounced by CD147 knockdown in the presence of TMZ (Figure 2E). Intriguingly, we identified the dramatic induction of ROS after TMZ treatment in glioma cells. The knockdown of CD147 further promoted TMZ-induced ROS production (Figure 2F). In accordance with the data in glioma cells with CD147 knockdown, CD147 overexpression increased cell growth rate and the resistance to TMZ treatment, and decreased TMZ induced ROS production, dose-dependently (Figure 2G-2K). Therefore, CD147 lead to the resistance of glioma cell to TMZ treatment potentially through elimination of TMZ induced ROS production.

### CD147 promotes Nrf2 expression through blocking its protein degradation

**Figure 3:**
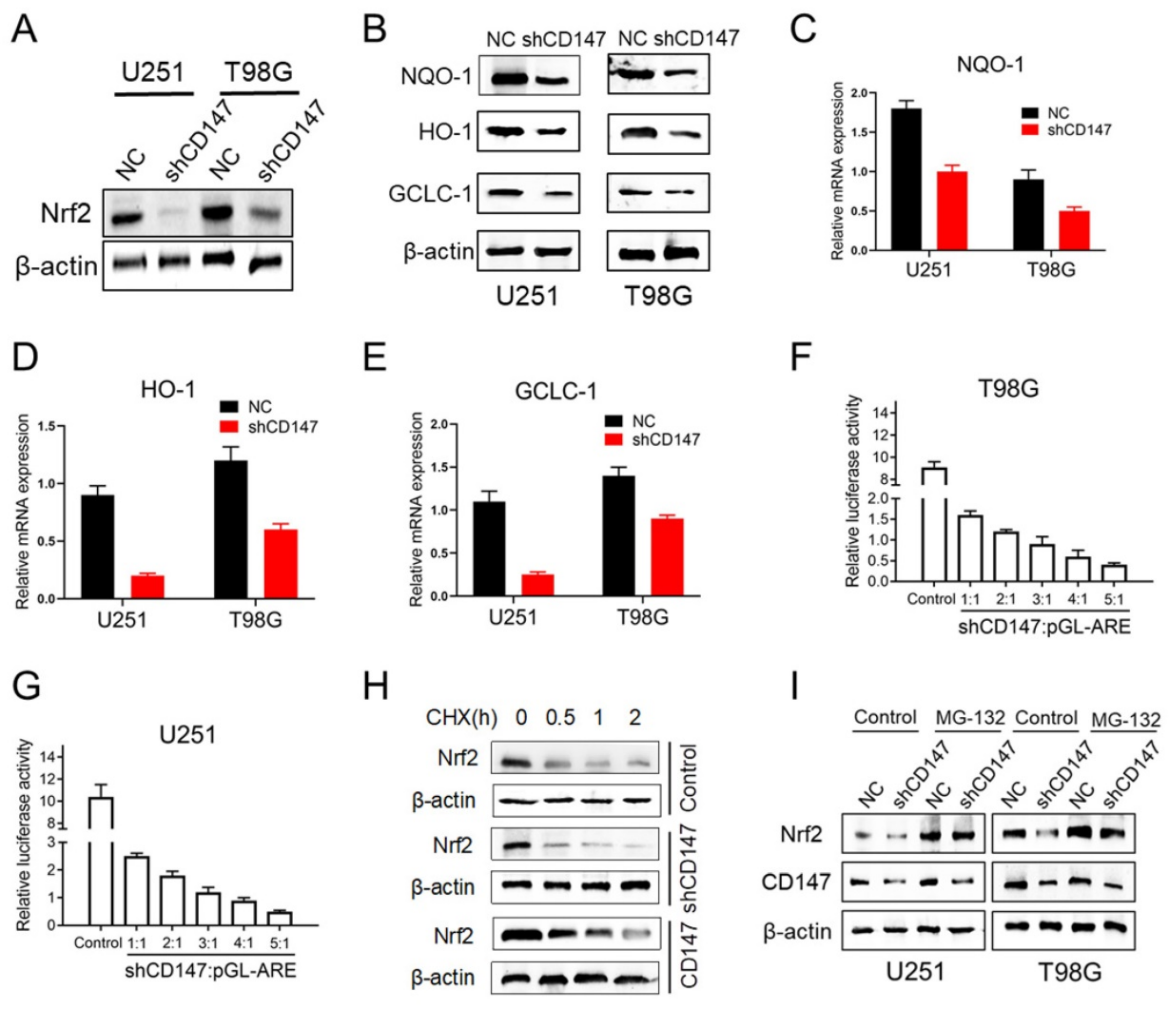
** CD147 promotes Nrf2 expression through blocking its protein degradation** (A) Nrf2 protein levels were determined in U251 or T98G cells after CD147 knockdown. (B) The indicated protein levels were determined in cells with or without CD147 knockdown. (C-E) NQO-1 (C), HO-1(D) and GCLC-1 (E) mRNA levels were determined by qCPR respectively. (F-G) Luciferase reporter gene assay was performed to determine CD147 knockdown could suppress the ARE activity. (H-I) Nrf2 protein stability were determined by western blotting in cells treated with CHX (10 μg/ml) for indicated time or MG-132 (10 μM) for 4 h, respectively.

To gain mechanistic insight into CD147-dependent drug resistance of glioma cells, we determined the genes involved in the regulation of redox homeostasis. Nrf2, which is a transcription factor that has been deemed the master regulator of cellular redox homeostasis, declined after CD147 knockdown (Figure 3A). NAD(P)H quinone oxidoreductase 1 (NQO1), Heme Oxygenase 1 (HO-1) and glutamate cysteine ligase catalytic subunit (GCLC-1), the key factors involved in the regulation of antioxidant process, were shown containing the anti-oxidative response element (ARE) in their promoters and transcriptionally activated by Nrf2 14. However, suppression of CD147 dramatically decreased NQO-1, HO-1 and GCLC-1 protein and mRNA expressions (Figure 3B-3E). Furthermore, CD147 shRNA suppressed ARE luciferase activity in a dose-dependent manner (Figure 3F and 3G). These data suggested that CD147 induces Nrf2 expression and its transcriptional activity, which may contribute to the elimination of intracellular ROS. As a transcription factor, Nrf2 activity and abundance are tightly regulated at post-translational level, such as phosphorylation, ubiquitination and acetylation, which are well characterized. The protein stability of Nrf2 could be regulated by ubiquitin-proteasome system, mediated by Keap1 or β-TrCP 14, 15. Here, we identified that Nrf2 stability was enhanced in the presence of CD147, and declined after suppression of CD147 (Figure 3H). In accordance, the decreased Nrf2 expression by CD147 knockdown were resumed after the treatment of proteasome inhibitor MG132 (Figure 3I). Therefore, CD147-dependent induction of Nrf2 was potentially through the blockage of Nrf2 protein degradation, and subsequently contributed to the elimination of TMZ induced ROS production.

### CD147 dictates Nrf2 stability through the suppression of GSK3β/β-TrCP dependent protein degradation

**Figure 4:**
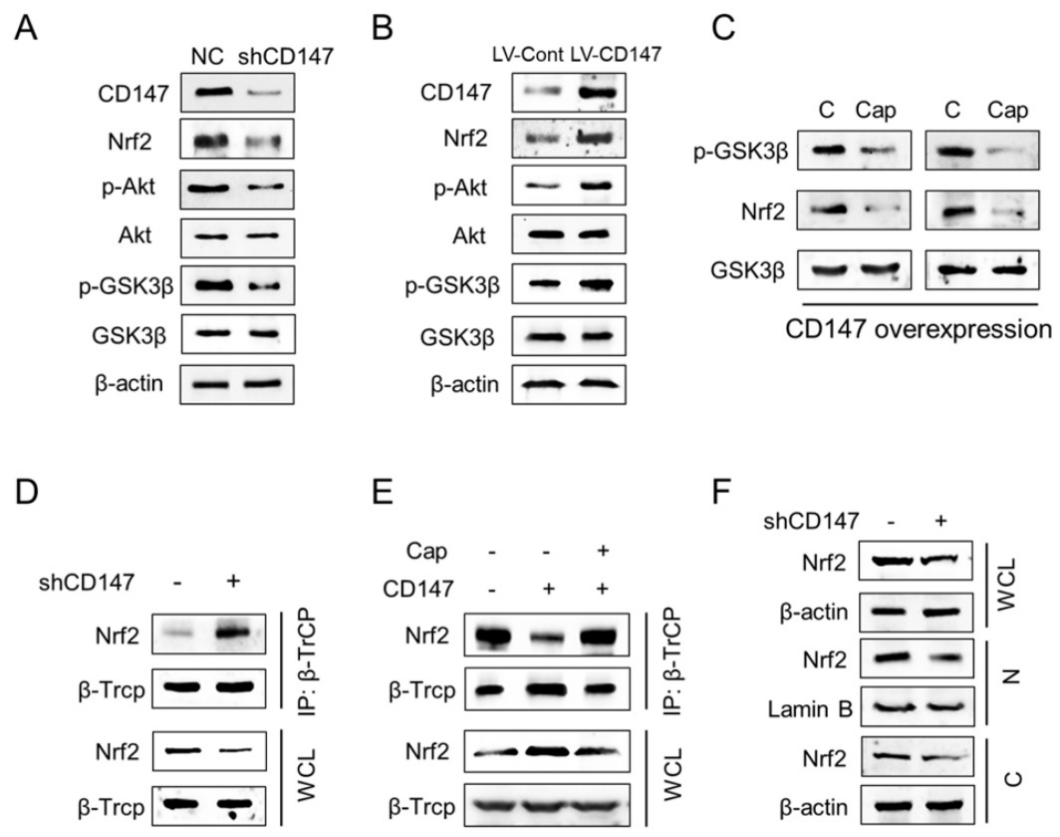
** CD147 dictates Nrf2 stability through the suppression of GSK3β/β-TrCP dependent protein degradation** (A-B) The indicated proteins were determined in U251 cells with CD147 knockdown (A) or overexpression (B). (C) Nrf2 and GSK3β protein levels were determined in U251 cells following Capivasertib (10nM) treatment. (D-E) Immunoprecipitation was performed to determine the interaction between β-TrCP and Nrf2 in indicated U251 cells. After the β-TrCP protein was immunoprecipitated with an anti-β-TrCP antibody, indicated proteins were detected by western blotting. (F) Suppresion of CD147 decreased the total and nuclear Nrf2 expression. β-Actin and Lamin B served as internal controls. WCL, whole cell lysate; N, nuclear; C, cytoplasmic.

Previous studies demonstrated that GSK-3β dependent phosphorylation of Nrf2 plays a key role in regulating Nrf2 stability. Phosphorylation of Nrf2 at Ser344 and Ser347 by GSK3β enhances the interaction between Nrf2 and E3-ligase β-TrCP, which results in the increased ubiquitylation and subsequent proteasomal degradation of Nrf2 16, 17. If GSK3β is inhibited by Akt or other enzymes, these Ser residues are dephosphorylated and Nrf2 can be released from β-TrCP, which allows nuclear translocation of Nrf2 14, 18. Thus we suppose CD147 may regulates Nrf2 expression through Akt/GSK3β axis. Expectedly, CD147 suppression by shRNA decreased phosphorylation of both Akt (Ser473) and GSK3β (Ser9) (Figure 4A), which were increased after CD147 overexpressed (Figure 4B). Accordingly, Nrf2 expression were decreased or increased, respectively, by CD147 knockdown or overexpression. Further, the treatment of Akt inhibitor Capivasertib decreased GSK3β (Ser9) phosphorylation and Nrf2 expression (Figure 4C). Thus, CD147 induced Nrf2 expression potentially through the activation of Akt and inactivation of GSK3β in advance. Especially, CD147 knockdown increased the interaction of CD147 with β-TrCP (Figure 4D). This interaction was decreased in the presence of CD147 and resumed after Capivasertib treatment (Figure 4E). Moreover, suppression of CD147 repressed Nrf2 protein levels in nuclear, suggesting that CD147 mediated inactivation of GSK3β/β-TrCP axis promotes Nrf2 stability and nuclear translocation (Figure 4F).

### CD147-dependent Nrf2 expression is required for glioma cells survival and drug resistance

**Figure 5:**
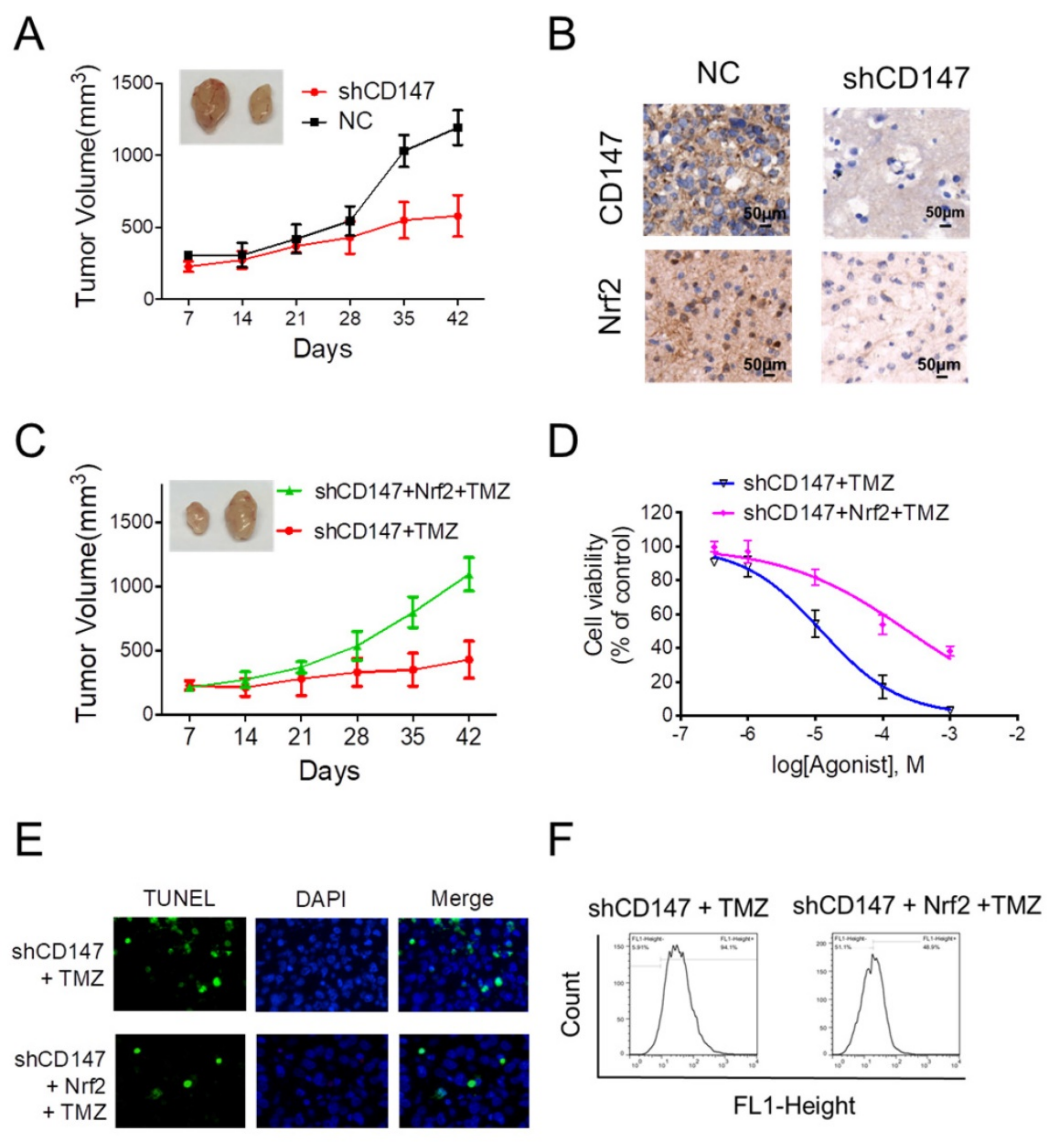
** CD147-dependent Nrf2 expression is required for glioma cells survival and drug resistance** (A) Suppression of CD147 increased anti-tumor effect of TMZ in nude mice. Mice were injected into the groin with 1 × 10^6^ U251 cells. The mice were given intraperitoneal injections of 50 mg/kg TMZ or DMSO once every day from days 1 to 5. The Tumor size was measured and tumor volume was calculated. (B) Immunohistochemistry staining of CD147 and Nrf2 in the sections of tumor graft. (C-F) Nrf2 levels were increased with lentivirus overexpressing Nrf2 in U251 cells with CD147 knockdown. (C-D) Nrf2 overexpression blocked TMZ mediated inhibitions of tumor growth in vivo (C) and in vitro (D). (E-F) Nrf2 overexpression blocked TMZ induced apoptosis (E) and ROS production (F).

Given the crucial function of Nrf2 on the regulation of neutralizing cellular ROS and restoring redox balance, we then sought to determine whether the dramatic alteration of Nrf2 levels in response to CD147 is functionally linked to CD147-triggered glioma cell survival and drug resistance. In the xenograft model, suppression of CD147 promoted the inhibitory effect of TMZ on glioma cell growth (Figure 5A). The reduced Nrf2 expression was also observed in the tumor section after CD147 knockdown (Figure 5B). We then restored Nrf2 expression in CD147 knockdown U251 cells. The presence of Nrf2 resumed the growth rate of U251 cells with TMZ treatment both in vivo and in vitro (Figure 5C and 5D). In accordance, Nrf2 overexpression suppressed TMZ-induced ROS production and cell death (Figure 5E and 5F). Therefore, CD147-dependent Nrf2 expression was a critical step for glioma cells with elevated CD147 expression resistant to TMZ treatment in clinical.

### CD147 and Nrf2 are positively correlated in glioma tissues and associated with patient outcome

**Figure 6:**
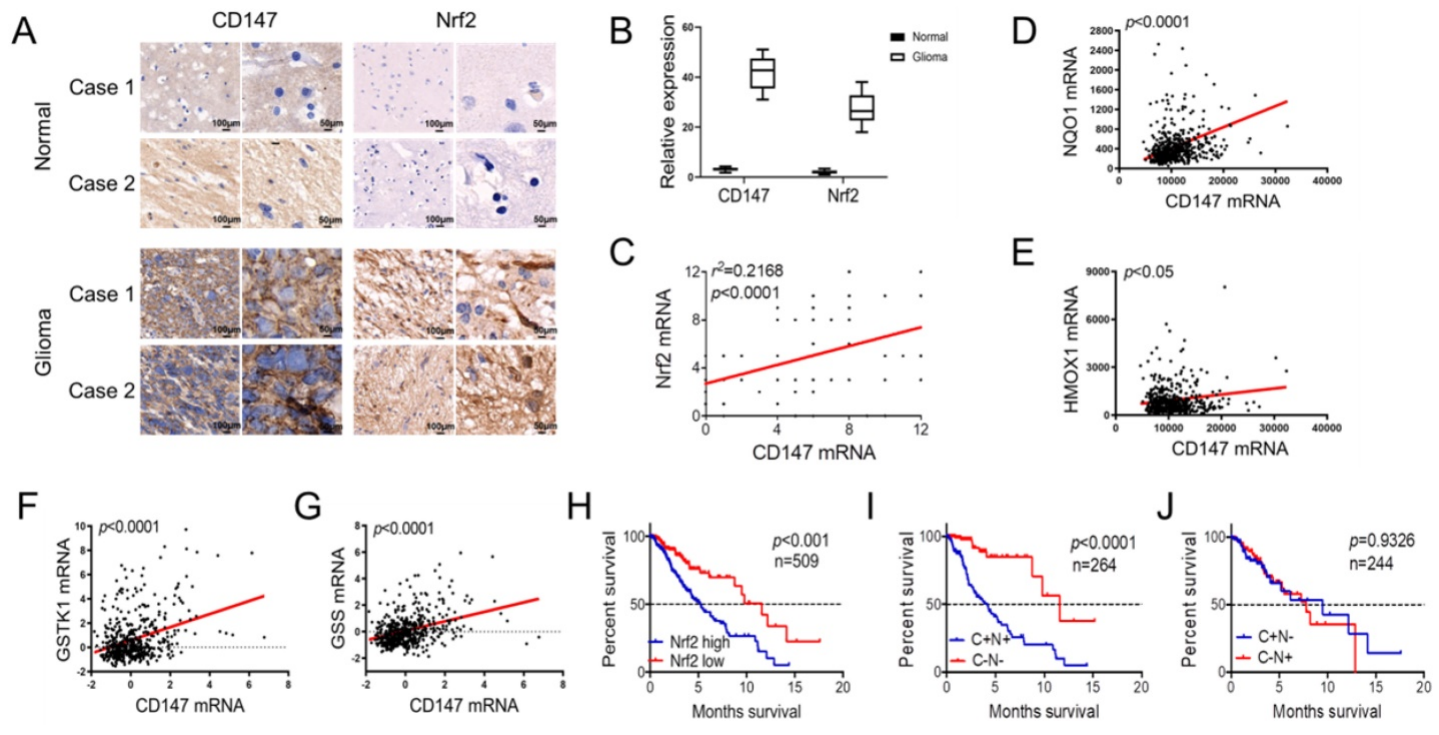
** CD147 and Nrf2 are positively correlated in glioma tissues and associated with patient outcome** (A-B) Immunohistochemistry staining of CD147 and Nrf2 in human adjacent normal and gioma tissues from patients (A) and the statistical analysis (B). (C) Positive correlation between CD147 and Nr2 expression levels with linear regression and Pearson's correlation significance (P < 0.0001, ANOVA test). (D-G) Positive association of CD147 with NQO-1 (D), HO-1 (E), GSTK-1 (F) and GSS (G) mRNA expression patterns in glioma tissues from TCGA data set by GEPIA with linear regression and Pearson's correlation significance. (H-J) Silico analysis of 509 cases of glioma tissues of the multidimensional data set from TCGA portal data set. KaplaneMeier plots indicate the clinical outcomes for Nrf2 (H), or CD147/Nrf2 levels (I and J) in glioma tissues. C, CD147; N, Nrf2; n, indicates the number of patient samples evaluated in each analysis. p-values were calculated using the ManteleCox log-rank test.

We further investigated the clinical significance of the reciprocal relationship between CD147 and Nrf2 in glioma and normal tissues. CD147 was broadly highly expressed in glioma tissues compared with normal brain tissues, and the expression pattern of Nrf2 was positive correlated with CD147 (Figure 6A-6C). Especially, in silico analysis of glioma tissues of the multidimensional data set from TCGA revealed that CD147 are broadly positive associated with Nrf2 target genes regulating redox homeostasis, including NQO-1, HO-1, GSTK-1, and GSS (Figure 6D-6G), which further confirmed the CD147 regulation of Nrf2. We demonstrated that high CD147 expression correlated with decreased survival (Figure 1G and 1H). Nrf2 expression also showed a significant power in terms of overall survival (Figure 6H). Especially, CD147-/Nrf2- gene signature was correlated with much better prognosis compared with CD147+/Nrf2+ gene signature (Figure 6I), whereas there were no significant differences between CD147+/Nrf2- and CD147-/Nrf2+ signature for patient survival (Figure 6J). Thus, the reciprocal regulatory relationship between CD147 and Nrf2 contributes to the aggressive phenotype generation and clinical outcome in glioma patients.

## Discussion

As a DNA alkylating agent, TMZ represents the most important chemotherapeutic option for glioma treatment. However, the therapeutic efficacy of TMZ remains very limited due to its frequent resistance in glioma, and the underlying mechanisms are complicated 19. Accumulated evidence identified that the acquisition of chemoresistance in gliomas is associated with the redox-balance, such as decreased ROS production 20, 21. In this study, we identified TMZ triggered ROS production is eliminated by Nrf2, which is induced by the elevated CD147 expression in glioma. Mechanism study shows that CD147 promotes Nrf2 protein stability through the suppression of GSK3β/β-TrCP dependent protein degradation via the activation of Akt pathway. Due to the reciprocal regulatory relationship between CD147 and Nrf2, which affects clinical outcome, targeting CD147 could suppress Nrf2 expression and further increase the anti-tumor effect of TMZ in glioma, validated by CD147 shRNA in this study.

CD147 is an oncoprotein highly expressed in multiple types of cancer. The chemotherapy resistant function of CD147 in various cancers was identified, but the related mechanism is complicated. The elevated CD147 expression in multiple myeloma cells lead to the resistance to immunomodulatory drugs, potentially through forming a complex with MCT1 in cell membrane 22. CD147 can also promote DNA damage response and gemcitabine resistance via targeting ATM/ATR/p53 and affects prognosis in pancreatic cancer 23. Especially, the high association of CD147 with redox hemostasis are also identified in numerus studies, but the underlying mechanism are not fully addressed 24, 25. We here identified that CD147 was highly expressed in glioma tissues and positively associated with tumor grade. The high levels of CD147 predicts a poor outcome potentially attribute to the TMZ resistance of glioma cells. Further, we identified that CD147 could suppress TMZ induced ROS production, which was associated with the induction of anti-oxidant protein Nrf2, which may provide a new clue to illustrate the mechanism of CD147-triggered drug resistance in malignant tumors.

Nrf2 is a nuclear transcription factor, and regulates the expression of downstream genes by recognizing ARE elements. Nrf2 regulates tumor progression through multiple ways. Firstly, Nrf2 enhances the antioxidant defense system of cells by inducing the synthesis of various antioxidant enzymes, such as HO-1, NQO-1 and GCLC-1, which are critical for the induction of Nicotinamide adenine dinucleotide phosphate (NADPH) and glutathione (GSH), contributing to anti-oxidative stress and promotion of tumor cell viability 5, 6. Secondly, Nrf2 induces tumor metabolic reprogramming by promoting pentose phosphate pathway (PPP). Nrf2 can transcriptionally induce the key enzymes of PPP, such as glucose-6-phosphate dehydrogenase (G6PD), 6-phosphate dehydrogenase gluconate (PGD), and further promote the production of nucleic acids NADPH for cell growth 26, 27. We here identified that CD147-dependent induction of Nrf2 played a key role in the regulation of redox homeostasis through eliminating ROS production, and subsequently promoteed the resistance of glioma cells to TMZ treatment. The antioxidant enzyme HO-1, NQO-1 and GCLC-1 induced by CD147 are Nrf2 targets containing ARE elements. Thus CD147 induced drug resistance is potentially through Nrf2-dependent induction of these antioxidant enzymes. The increased ARE luciferase activity by CD147 shRNA also confirmed this idea.

Based on the key function of Nrf2 on redox hemostasis and drug resistance in tumor cells, the factors involved in regulating Nrf2 expression were identified. Keap1 and β-TrCP are the key modulators for the post-translational regulation of Nrf2 expression, in response to different pathways 8, 17, 28, 29. β-TrCP serves as the substrate recognition subunit for the SCF/β-TrCP E3 ubiquitin ligases, which ubiquitinate specifically phosphorylated substrates and play a pivotal role in the regulation of cell division and various signal transduction pathways, which are essential for many aspects of tumorigenesis 30. Nrf2 has shown that downstream of GSK-3β, and the phosphorylated Nrf2 by GSK-3β could be recognized by β-TrCP, resulting in Nrf2 protein degradation 9. Here, we found that the elevated CD147 in glioma cells suppressed GSK-3β activity, which in turn blocked SCF/β-TrCP mediated Nrf2 protein degradation. Thus our study identified a new clue of CD147 mediated Nrf2 expression through post-translational regulation. However, we did not determine whether Keap1 can also regulate Nrf2 stability in response to CD147. Further, Nrf2 mRNA levels were also slightly decreased upon CD147 shRNA treatment (data not shown). Thus, we suppose CD147 regulates Nrf2 through multiple pathways. Further studies are needed to fully address the related mechanism.

Based on the reciprocal regulatory relationship between CD147 and Nrf2 identified in this study, we further correlated the expressions in glioma patients. The expression pattern of CD147 was positive correlated with Nrf2 and its target genes, confirming our proposal of CD147-dependent induction of Nrf2 in glioma cells. Further, based on the TCGA analysis, the increased CD147 or Nrf2 levels appear to be a relatively early event in tumorigenesis and correlate with worse prognosis later in tumorigenesis (Figure 1G and 6G). Noticeably, we found that an CD147-/Nrf2- gene signature was correlated with much better prognosis compared with CD147+/Nrf2+ gene signature, that meant the negative effect of increased CD147 expression was most evident with increased Nrf2 levels. Thus we verified the clinical significance of CD147-Nrf2 axis in glioma progression and drug resistance, and targeting both CD147 and Nrf2 were necessary for the treatment of glioma patients.

In summary, our data provide the first evidence that glioma resistance to TMZ is potentially due to the activation of CD147/Nrf2 axis. CD147 promotes Nrf2 stability through the suppression of GSK3β/β-TrCP dependent Nrf2 protein degradation, which results in the ablation of TMZ induced ROS production. As such, we point out that targeting CD147/Nrf2 axis may provide a new strategy for the treatment of TMZ resistant gliomas.

## Materials and Methods

### Cell culture and conditions

The human glioma cell line U251 was purchased from the Cell Bank of the Type Culture Collection of the Chinese Academy of Sciences, Shanghai, China. The human glioma cell line T98G was purchased from the China Center for Type Culture Collection, Wuhan, China. All of the cell lines were authenticated by short tandem repeat DNA profiling. They were cultured in DMEM (GIBCO) with 10% fetal bovine serum (FBS) at 37°C in a 5% CO_2_ humidified atmosphere.

### Virus and reagent

CD147 overexpression and knockdown lentivirus was packaged in JiKai Biotechnology, Shanghai, China. MG132 (HY-13259) and Temozolomide (HY-17364) were purchased from MedChemExpress. Capivasertib (S8019) was purchased from Selleck.cn.

### Immunoprecipitation and Western blotting

Cells were harvested and lysed. The lysate was centrifuged, then incubated with anti-β-TrCP antibody (#4394, Cell signaling Technology) overnight at 4°C. For each IP sample, 500 µl of the lysate was incubated overnight at 4°C with 1 µg of the indicated antibody and 10 µl of Protein A/G Plus-Agarose (sc-2003, Santa Cruz). After incubated with Protein A/G Plus-Agarose for 1h on a rocking platform, the complex was washed three times with cold IP buffer and 1 X SDS loading buffer was added. Finally the whole-cell lysates and immunoprecipitated proteins were subjected to Western blotting analysis. Western blotting assay was performed as previously described. Primary antibodies were used at dilutions of 1:1000 for anti-CD147 (ab108308, Abcam), anti-Nrf2 (ab180845, Abcam), anti-NQO-1 (ab28947, Abcam), anti-HO-1 (ab13248, Abcam), anti-GCLC-1 (ab190685, Abcam), anti-β-TrCP (#4394, Cell signaling Technology), anti-Phospho-GSK-3-beta (Ser9) (#9322, Cell Signaling Technology), anti-GSK-3-beta (#12456, Cell Signaling Technology), anti-Phospho-Akt (Ser473) (#4051, Cell Signaling Technology), anti-Akt (#9272, Cell Signaling Technology), and anti-β-actin (#A5441, Sigma) antibody, anti-Lamin B(#13435, Cell Signaling Technology), respectively.

### Quantitative PCR

Total RNA was extracted and used to cDNA synthesis through PrimeScript™ RT Master Mix (RR036A, Takara). TB Green® Fast qPCR Mix (RR430A, Takara) was used to detect the PCR amplification products. The quantitative PCR (qPCR) assay was performed as described previously 31. Primer sequences are available on request.

### Tissue samples and immunohistochemistry staining

This study was approved by the ethics committee of the Fourth Military Medical University. Glioma tissue microarray was purchased from the pathology department of the Fourth Military Medical University. A total of 70 glioma samples were collected, including 6 para-carcinoma tissue, 13 stage I glioma, 22 stage II glioma, 16 stage III glioma, 13 stage IV glioma. The immunohistochemistry staining and analysis were performed as described previously 32. Student's t-test was applied for statistical analyses of the relative protein levels. The linear regression and Pearson's correlation significance were used to analyze the correlation between CD147 and Nrf2 expressions.

### EdU proliferation assay

To assess cell proliferation, cells were seeded in 24-well plates with complete media. Cell proliferation was detected using the incorporation of 5-ethynyl-20 -deoxyuridine (EdU) with the EdU Cell Proliferation Assay Kit (Ribobio, Guangzhou, China). Briefly, the cells were incubated with 50 mM EdU for 6 h before fixation, permeabilization and EdU staining, which were performed according to the manufacturer's protocol. The cell nuclei were stained with DAPI (Sigma) at a concentration of 1 mg/ml for 20 min. The proportion of the cells incorporated EdU was determined with fluorescence microscopy.

### Cell Counting Kit-8 (CCK-8) Assay

Cells were seeded at a density of 1000 cells/well in 96-well plates and had been incubated in a 5% carbon dioxide incubator at 37°C overnight. Cells treated with DMSO and TMZ for as indicated time. Then, 10 μL of CCK-8 solution (Targetmol C0005, China) were added to each well and incubated with the cells for 4 hours at 37°C. Lastly, the absorbance was measured at 450 nm using a spectrophotometer.

### ROS detection and TUNEL Assay

TUNEL using an One Step TUNEL Apoptosis Assay Kit (Beyotime C1086, China) according to the manufacturer's instructions as described previously 33. ROS levels in cells were measured by flow cytometry with the ROS Assay Kit (Beyotime S0033, China) as described previously^33^.

### Protein degradation assay

Cells were incubated with CHX (10 μg/ml, #2112, Cell Signaling Technology) for the indicated time. Cells were then harvest and western blotting was performed as described above.

### *In vivo* tumorigenicity assay

To assess subcutaneous tumor growth, U251 and U251-shCD147 cells (1×10^6^) in 150 μL of PBS were injected into the groin of the eight-week-old male nude mice. The mice were given intraperitoneal injections of 50 mg/kg TMZ or DMSO once every day from days 1 to 5. Tumor volumes were measured one time per week with a caliper using the formula V = π × [d^2^×D]/6, where d is the minor tumor axis and D is the major tumor axis.

### TCGA patient data analysis

The Cancer Genome Atlas data was used to determine the patient survival and the tendency for cooccurrence of CD147, Nrf2, NQO-1, HO-1, and GSTK-1 alterations in the data set for Low Grad Glioma (LGG). The online analysis tool UALCAN, PROTEINATLAS and TCGAportal were used for CD147 expression and survival analysis. CD147(BSG), Nrf2(NFE2L2), NQO-1, HO-1, and GSTK-1 were entered as the query genes and overall survival and co-expression was provided through the relative user interface. CD147 protein expression and copy numbers were determined in various types of tumor sections by PROTEINATLAS. Clinical outcomes for CD147 gene expression patterns were determined by UALCAN analysis. The raw data were downloaded from TCGA through the TCGAportal and manually graphed in Prism (Graphpad) with the mRNA alterations and clinical outcomes for CD147 and Nrf2. The Gene Expression Profiling Interactive Analysis (GEPIA) was used to determine the correlation of CD147 with Nrf2 target genes in the dataset of LGG. For survival analysis, Kaplan-Meier survival graphs were plotted, and log-rank tests were performed.

### Statistical Analysis

Data are expressed as mean±SD. Statistical analysis was performed with the SPSS10.0 software package by using student's T test for independent groups. Statistical significance was based on a value of P≤0.05.

## Abbreviations

TMZ: temozolomide
ROS: reactive oxygen species
β-TrCP: β-transducin repeats-containing proteins
SCF: Skp1-Cul1-F-box-protein
CD147/BSG: basigin
TUNEL: TdT-mediated dUTP Nick-End Labeling
Nrf2: nuclear factor E2-related factor 2
